# The short-term effects of a mass reach physical activity campaign: an evaluation using hierarchy of effects model and intention profiles

**DOI:** 10.1186/s12889-018-6218-7

**Published:** 2018-11-27

**Authors:** T. R. Berry, R. E. Rhodes, E. M. Ori, K. McFadden, G. Faulkner, A. E. Latimer-Cheung, N. O’Reilly, J. C. Spence, M. S. Tremblay, L. M. Vanderloo

**Affiliations:** 1 Faculty of Kinesiology, Sport, and Recreation, 1-153 University Hall; University of Alberta, T6G 2H9, Edmonton, AB 780 492 3280 Canada; 2 University of Victoria, Victoria, Canada; 3 University of British Columbia, British Columbia, Canada; 4 Queen’s University, Kingston, Canada; 5 University of Guelph, Guelph, Canada; 6 Children’s Hospital of Eastern Ontario Research Institute, Ontario, Canada; 7 ParticipACTION, Ontario, Canada

**Keywords:** Physical activity, Mass media campaigns, Evaluation, Hierarchy of effects, Intention profiles, Post-campaign effects

## Abstract

**Background:**

Mass reach physical activity campaigns are designed to deliver physical-activity related messages to a large population across different media including print, television, radio, and websites. Few evaluations have examined the short-term effects of a mass reach campaign on participants who were engaged with the campaign. The current research examined the short-term effects of the ParticipACTION 150 Play List, a mass reach physical activity campaign, on participants who registered with the campaign website.

**Methods:**

Participants (*N* = 7801) completed a registration questionnaire measuring demographic information, awareness and recall of physical activity and sport advertising, and self-reported number of activities tried or planned to try from the 150 Play List. A follow-up survey was completed by 1298 participants from the original sample. Additional questions assessed experience with the 150 Play List and attitudes towards campaign advertisements.

**Results:**

Approximately 14.5% of participants cited the ParticipACTION 150 Play List and 23.6% mentioned a ‘getting active’ message when recalling advertisements. Those who named the 150 Play List or getting active reported more activities tried and more activities planned than those who did not. They were also more likely to say they had tried a new activity and planned ongoing participation. It was also found that participants with a disability were more likely to have tried a new activity compared to those not in a minority group. Other correlates of trying new activities at follow-up were younger age, more positive reported experience with the 150 Play List, and more favourable attitudes towards campaign advertisements. Those who did not intend continued participation, or who were unsure at baseline and then decided against continued participation at follow-up, reported they were less sedentary or encouraging others to be active.

**Conclusions:**

This research addresses the gap in evidence regarding the efficacy of mass reach physical activity campaigns by informing whether a year-long campaign like the 150 Play List can be effective in influencing the behavior of those engaged with the campaign. The results reinforce the idea that ‘top of mind’ awareness should be measured. Investigating intention profiles can help inform campaign impacts and continuation intentions.

**Electronic supplementary material:**

The online version of this article (10.1186/s12889-018-6218-7) contains supplementary material, which is available to authorized users.

## Background

Mass reach physical activity (PA) campaigns are designed to deliver PA-related messages to a large population across different media including print, television, radio and websites. A review indicates that most evaluations of such campaigns measure intentions rather than behaviors as the outcome [1]. Others have similarly highlighted that short-term outcomes, such as changes in attitudes and intentions, are more often measured [2]. Most evaluation studies included in these reviews examined campaign effects across a population, such as how many people in the target audience were aware of the campaign and subsequently affected by it. Few evaluations have examined the effects of a mass reach campaign on participants who were engaged with the campaign; in other words, whether PA participation that can be in part attributed to the campaign, is maintained. The current research examined the short-term effects of the ParticipACTION 150 Play List, a mass reach PA campaign (referred to as the 150 Play List hereafter), on participants who registered with the campaign website.

ParticipACTION, a Canadian, national not-for-profit organization with the mandate of promoting PA, was selected by Heritage Canada (the Canadian governmental department responsible for policies and programs regarding the arts, culture, media, and sports) to create one of the signature initiatives in celebration of Canada’s 150th birthday in 2017. The resulting campaign was the 150 Play List [3]. In its first phase (October 12 – December 9, 2016), Canadians were invited to suggest physical activities, that any person might ostensibly try, for possible inclusion. These activities, both sport (e.g., ball hockey, sitting volleyball) and non-sport (e.g., walking, gardening), were voted on by the Canadian public. ParticipACTION created a final list of 150 activities that reflected the most popular activities, while also ensuring activity diversity and inclusivity (i.e., all activities had to be suitable for adaptation for people with a sensory, intellectual, or mobility disability). In addition, seven traditional Indigenous sports (e.g., knuckle hop) were included. The 150 Play List was launched in January 2017 and each of the final 150 activities had a feature page on the campaign website. ParticipACTION also hosted 100 community events across Canada throughout the year with the intent of attracting Canadians to try some or all of the activities. The website was produced in both English and French and community events were hosted in both languages. Canadians, aged ≥13 years, could track their completed activities on the website and be eligible for prizes. There were campaign advertisements on national television and radio, and social media. In addition, a media sponsor profiled activities and events during news broadcasts.

The hierarchy of effects model (HOEM) [4] was used to guide this evaluation of the 150 Play List. The HOEM proposes that evaluations of mass reach campaigns should first establish if there are immediate effects, such as awareness of the campaign, prior to evaluating more distal goals including changes in attitudes, self-efficacy, or intentions, and ultimately behavior [4]. Intuitively, engaging with a program component would suggest awareness (e.g., registering as a program participant). However, research has found that among participants who registered on a PA campaign website, those who named the campaign without prompting (only 59% of the study sample) reported higher attentional bias toward campaign logos and higher self-reported PA compared to those who did not name the campaign. [5]. As these authors argue, not naming the campaign by website registrants indicates that campaign evaluators should recognize that people registered with a campaign may not actually be thinking much about it. Thus, the current research similarly assessed ‘top of mind’ campaign awareness among campaign registrants, in addition to other HOEM variables: attitudes, self-efficacy, and intentions, and behavioral outcomes such as trying new activities. The HOEM has been used to examine population effects across many mass reach campaigns (cf. [1, 2]) including others from ParticipACTION [6, 7], although some studies with other campaigns have found limited support for the sequence of effects outlined in the model [8]. Regardless of whether the cascade of effects occurs in sequence (e.g., change in attitudes precedes behavior change), using the HOEM can guide evaluation of different levels of effects.

In addition to the HOEM, the current research also examined campaign effects across profiles of non-intenders (those who have no intention of continued participation in 150 Play List activities), ambivalent intenders (those who are unsure if they will continue participation), and intenders (those who intend to continue to participate). This is a novel contribution to evaluation research because it addresses the intention-behavior gap, where roughly half of all people with positive intentions to engage in PA do not act on them [9]. The separation of intention-behavior profiles is known as an action control framework, and is useful for exploring the predictors of intention formation and the translation of intentions into behavior [10]. The current research focused on intention to continue with 150 Play List activities, as these continuation intentions are an important determinant of maintained behavior [11].

Therefore, the purpose of this research was to investigate the short-term effects of the 150 Play List among participants who registered for the campaign using the HOEM as a guide and across different intention profiles.

## Methods

### Participants and procedure

Every person ≥13 years who registered online with the 150 Play List (*N* = 81,113) was sent an invitation to complete the registration questionnaire. The survey was not implemented until after the campaign started but participants already registered were sent the invitation. The registration survey data were collected from June through November 2017. Participants were asked if they would be willing to complete another survey in the future and if so, to provide their first and last names, and e-mail address. Those who did so were sent an invitation to complete the follow-up survey after the campaign ended in December 2017. Follow-up data were collected from January 15 to February 25 2018. Surveys were sent in both English and French, based on participant’s preference. Both surveys received ethical approval from a university human research ethics board. Informed consent was indicated by starting the survey.

### Surveys

The survey questions are shown in the Additional file 1. The registration survey included questions on demographics, awareness, and 150 Play List participation (i.e., number of activities from the 150 Play List tried or planned, and the number of new activities tried, or planned). The participation questions were developed by ParticipACTION for their evaluation and others have used questions about planned activities as a measure of ongoing intention [11, 12]. Questions about planning and ongoing participation can be considered as forms of continuation intentions [11]. After these items, participants < 18 years were shown Canada’s PA guidelines for children and youth (i.e., at least 60 min of moderate to vigorous PA per day). Participants ≥18 years were shown Canada’s PA guidelines for adults (i.e., at least 150 min of moderate to vigorous PA per week). Items regarding PA importance, attitudes, self-efficacy, and intentions (i.e., proximal HOEM constructs) were asked according to the age-specific guidelines. Previous research has supported the use of single item measures when they have good construct validity [13]. The LTPA item has been validated using doubly-labelled water and advocated for use in population level studies [14]. The follow-up survey included information about 150 Play List participation, importance, attitudes, self-efficacy, intentions, and experience with the 150 Play List. Attitudes toward 150 Play List advertisements were also assessed. For that scale, the ‘boring’ and ‘not realistic’ items were reverse-scored, and the inter-rater reliability across all items was good, α = .85, therefore a mean score for attitudes was created.

### Data analysis

Names and email addresses were matched across the two surveys and birth year was cross-referenced with the birthdate the participant provided when registering on the 150 Play List website. After data were matched, all identifying information was removed from the data set to comply with ethics. Data were cleaned first by removing cases where two or fewer survey items were responded to and when reported age was < 13 years (the minimum response age) or when the number of 150 Play List activities reported was > 150. Missing data in the remaining surveys were mean replaced (continuous variables). Missing sex and education were recoded as ‘prefer not to answer’, missing minority group data were recoded as no minority group, and missing LTPA data were recoded as ‘moderate’. Analyses using these data were compared to the results of analyses conducted on surveys with no missing data. Alpha was set to .01 for all analyses and Cohen’s *d* reported for between group effect sizes.

### Key messages

The responses from the baseline awareness question “Can you describe any of the key messages?” were first open coded by one researcher who also wrote code descriptions. After initial codes were generated, three researchers (all of whom are bilingual in English and French) discussed them and agreed on the final codes. A random sample of 1/3 of the responses was selected using SPSS (version 24) and a second researcher coded those using the code descriptions to determine inter-rater reliability. Any discrepancies were discussed to assign final codes to responses. Some responses had up to four different codes embedded within them. For example, the response “get outside, get moving” received two codes – ‘get active/fit’ and ‘get outdoors’. The first rater also created a subcode for the ‘PA (specific names)’ code. If a specific activity was mentioned (e.g., walking) then it was given a subcode to determine how many times a specific activity was mentioned. Again, multiple subcodes could be given to one response. For example, the response “Articles about walking, running, weight lifting” received three subcodes to reflect the named activities.

### Baseline participation

A binomial logistic regression was conducted to predict whether participants reported trying new activities (yes/no). Linear regressions were used to predict the numbers of activities from the 150 Play List: tried since registration, planned to continue, new activities tried, and new activities planned to continue. In all models, demographic information (age, sex, education, minority group, LTPA) was entered in the first step, whether the 150 Play List or getting active in responses to key messages was mentioned (i.e., top of mind awareness) were entered into the second step, and importance, attitudes, self-efficacy, and intentions in the third. Using the same variables, a multinomial logistic regression was constructed to examine whether participants planned ongoing participation (yes/no/not sure).

### Follow-up

Intention groups were formed based on the question asked at baseline and follow-up, “Did any of the activities you tried on the 150 Play List result in on-going participation?” The intention groups formed were successful intenders (reported ongoing participation at both assessment points), non-intenders (no ongoing participation at either assessment point), unsuccessful intenders (reported ongoing participation at baseline but not at follow-up), disinclined actors (reported no ongoing participation at baseline, but did at follow-up), ambivalent nonactors (were not sure at baseline and reported no ongoing participation at follow-up), ambivalent actors (were not sure at baseline but did report ongoing participation at follow-up), and ambivalent (unsure at both assessment points) groups were compared to successful intenders. Repeated measures analysis of variance tests (RM ANOVAs) were used to examine changes over time in number of activities tried since registration, importance, attitudes, self-efficacy, and intentions with baseline and follow-up scores as the within subjects variables and intention group as the between subjects variable. Chi-square analyses were calculated to determine differences in intention groups in the impact of the 150 Play List (e.g., increased PA participation).

A binomial logistic regression was conducted to predict whether participants reported trying new activities (yes/no). Logistic regressions were used to predict the number of activities from the 150 Play List tried since registration and the number of activities participants plan to try. In all these models, demographic information was entered in the first step, whether they mentioned the 150 Play List or getting active in response to key messages at baseline were entered into the second step, and importance, attitudes, self-efficacy, intentions (all baseline measures), experience with the 150 Play List, and attitudes toward the 150 Play List advertisements were included in step 3. A multinomial logistic regression using the same variables was constructed to predict intention group (non-intenders, unsuccessful intenders, disinclined actors, ambivalent nonactors, ambivalent actors, and ambivalent groups were compared to successful intenders).

## Results

A total of 10,124 people started the baseline registration survey but data from 2248 were removed because they responded to two or fewer of the survey items. Data from 74 participants were removed because they reported their age as < 13 years and one reported > 150 activities completed. This left 7801 cases at baseline (*n =* 7292 English, *n =* 509 French). Of these, 6824 had no missing data. Missing data ranged from *n* = 48 [importance] to *n* = 184 [intentions]). Other missing data included sex (*n* = 5), education (*n* = 7), minority group (*n* = 450), and LTPA (*n =* 127). At follow-up, 1237 English language and 61 French language surveys, matched to registration data for within-subjects comparisons, were completed from a possible 1664. Though no demographic data were available for the unmatched follow-up surveys, no differences existed in the number of activities tried or planned, nor in any social cognitive variables between the 1298 matched to baseline and the 366 unmatched follow-up surveys (all *p* > .09). Table 1 shows the demographic characteristics of those who completed only the baseline survey compared to those who completed both the baseline and follow-up survey. The difference in time between completing baseline and follow-up surveys ranged from 35 to 229 days (*M* = 158.8 [SD = 45.33]). No differences existed in time between surveys between any demographic or intention groups (all *p* > .15), nor was time between surveys correlated with any attitudes, self-efficacy, intentions, or activities tried or planned variables (all *r* < .09).

**Table 1:** Demographic information and differences between participants who completed follow-up and those who did not

		Baseline only (*N* = 6503)	Complete follow-up (*N* = 1298)	Difference test
Female N (%)		5322 (81.8%)	1048 (80.7%)	χ^2^ = 1.63, *p* = .44
Minority N (%)	No	5484 (84.3%)	1102 (84.9%)	χ^2^ = 11.68, *p* = .02 More new Canadians and Indigenous persons did not complete follow-up survey than expected.
	Visible	416 (6.4%)	91 (7.0%)	
	New Canadian	158 (2.4%)	15 (1.2%)	
	Indigenous	140 (2.2%)	20 (1.5%)	
	Disability	305 (4.7%)	70 (5.4%)	
English N (%)		6055 (93.1%)	1237 (95.3%)	χ^2^ = 8.51, *p* = .004 More English than French completed follow-up
Education N (%)	Some high school	153 (2.4%)	14 (1.1%)	χ^2^ = 13.89, *p* < .02 Slightly more educated completed follow-up
	High school	591 (9.1%)	124 (9.6%)	
	Some college	1000 (15.4%)	183 (14.1%)	
	University	3313 (50.9%)	687 (52.9%)	
	Post degree	1325 (20.4%)	275 (21.2%)	
	Prefer not to answer	121 (1.9%)	15 (1.2%)	
LTPA N (%)	Very light	121 (1.9%)	14 (1.1%)	χ^2^ = 7.89, *p* = .10
	Light	611 (9.4%)	139 (10.7%)	
	Moderate	2241 (34.5%)	431 (33.2%)	
	Active	2420 (37.2%)	506 (39.0%)	
	Very active	1110 (17.1%)	208 (16.0%)	
Age M (SD), years		47.84 (13.43)	48.62 (12.58)	F (1, 7800) = 3.72, *p* = .06
Importance M (SD)		6.22 (1.08)	6.22 (1.07)	F (1, 7800) = 0.038, *p* = .85
Attitudes M (SD)		5.49 (1.57)	5.54 (1.53)	F (1, 7800) = 2.78, *p* = .29
Self-efficacy M (SD)		81.77 (21.87)	82.01 (22.03)	F (1, 7800) = 0.13, *p* = .72
Intention M (SD)		6.01 (1.30)	5.97 (1.35)	F (1, 7800) = 1.54, *p* = .34

### Key messages

A total of 4201 open-ended responses were provided in English (*n* = 4012) and French (*n* = 189). The first coder generated an initial 14 codes and after discussion 12 codes were agreed on. Inter-rater reliability was good, kappa = .84, *p* < .001. Most of the discrepancies were in the ‘150 Play List/activities’ code. Many of these were simply in the order the code was assigned when multiple codes were given to one response. Discrepancies were discussed and final codes assigned. Table 2 shows the final codes, examples of statements within these codes, and the number of times each was mentioned. Within the ‘PA (specific names)’ code, 81 unique activities or sports were mentioned. Those most often mentioned were walking (*n* = 39), running (*n* = 34), biking (*n* = 28), yoga (*n* = 24), and skating (*n* = 19). Hockey was the sport most often mentioned in one of its various forms: ice, ball, street, or sledge (*n* = 27).

**Table 2:** Final key message codes and the number (%) of baseline and follow-up respondents who gave corresponding responses

Code Name	Sample Response	Baseline N (%)	Follow-up N (%)
150 Play List/activities	Join the ParticipACTION play list	1133 (14.5)	204 (15.7%)
Canada 150 celebration	It had to do with Canada’s 150th birthday	328 (4.2)	53 (4.1%)
Get active/fit	Getting active, getting involved in activities with others	1841 (23.6)	319 (24.6%)
Get outdoors	Get outside, play, run, sweat	475 (6.1)	93 (7.2%)
Physical activity (specific names)	Sledge hockey, street hockey, dancing	657 (8.4)	119 (9.2%)
Try something new	Try a new sport	308 (3.9)	57 (4.4%)
Location of ad specified	Global morning news	403 (5.2)	71 (5.5%)
Health benefits	Good for your health, heart and mind	432 (5.5)	75 (5.8%)
Affective response	Have fun participating	409 (5.2)	85 (6.5%)
PA recommendations	Get 150 min of physical activity	51 (0.7)	6 (0.5%)
Other advertisements catch phrase	The Hal & Joanne Get Fit commercials are what comes to mind	118 (1.5)	21 (1.6%)
Miscellaneous and irrelevant	Humans are amazing	263 (6.6)	(1.8%)

### Baseline predictors of participation

The results of the regression analyses are shown in Table 3. All models accounted for very small proportions of variance, but significant correlates of increased participation included mentioning the 150 Play List or ‘getting active’, importance, attitudes, and intentions.

**Table 3:** Step 3 F-tests and coefficients for the baseline linear regression models (number of activities tried and plan to try), logistic regression model (tried new activity), and multinomial logistic regression model (plan ongoing participation)

	Number of activities tried	Number planning to try		Tried new activity (compared to no)	Plan ongoing participation (compared to no)	
Descriptives	*N* = 7777, *M* = 21.98 (*SD* = 28.52)	*N* = 7217, *M* = 47.99 (*SD* = 48.88)		Yes *n* = 4410, No *n* = 3391	Yes *n* = 3697, No *n* = 1688, Not sure *n* = 2416	
	F (4, 7789) = 23.17, *p* < .001; R^2^ = .037	F (4, 7789) = 43.42, *p* < .001; R^2^ = .076		Χ^2^ = 228.62 (*df* = 20), *p* < .001; Nagelkerke R^2^ = .039	Χ^2^ = 367.95 (*df* = 40), *p* < .001; Nagelkerke R^2^ = .053	
			Categorical predictor categories	Yes	Yes	Not sure
Predictor	Beta	Beta		Exp (B)	Exp (B)	Exp (B)
Age	.013	−.201***		.991**	1.01**	1.00
Sex	.068**	.014	Prefer not to answer	.888	0.414	1.172
			Female	.853	0.827	0.951
			Male ^a^	–	–	–
Education	.014	−.055***	Prefer not to answer	.992	1.970	1.767
			High school or less	1.096	1.558**	1.121
			Some college	1.211	1.199	1.174
			College/ university degree	1.038	1.082	1.031
			Postdegree ^a^	–	–	–
Minority group	−.024	.014	Visible minority	1.251	1.376	1.010
			New Canadian	1.674*	1.820*	1.162
			Indigenous	.958	1.795	1.802
			Disability	1.616**	1.437	1.374
			No ^a^	–	–	–
LTPA	.054**	.002	Very light or light	.925	1.186	1.786**
			Moderate	1.190	1.640**	2.013**
			Active	1.012	1.408**	1.526**
			Very active^a^	–	–	–
Mentioned 150 Play List	.044**	.074**		1.52**	1.351**	1.151
Mentioned ‘getting active’	.060**	.053**		1.410**	1.466**	1.357**
Importance	.064**	.058***		1.106**	1.113**	1.005
Affective attitudes	.089**	.125***		1.072**	1.038	1.045
Self-efficacy	−.005	−.022		.994**	.993***	.994*
Intentions	−.006	.033		1.096***	1.305***	1.137**

^a^comparison group; * *p* < .01, **p < .001

### Follow-up analyses

All reported analyses were conducted with the original data and with missing data replaced. No differences were observed in any of the analyses and the results reported are those with missing data replaced.

### Differences by intention group

The seven intention groups included 426 successful intenders, 112 non-intenders, 111 unsuccessful intenders, 93 disinclined actors, 145 ambivalent nonactors, 258 ambivalent actors, 153 classified as ambivalent. No differences were observed in sex, education, or minority group by intention group. Successful intenders were significantly older (M = 50.73 yrs. [SD = 12.05]) than non-intenders (M = 45.70 yrs. [SD = 13.06]), and unsuccessful intenders (M = 45.12 yrs. [SD = 13.01]); all *p* < .01. Differences in LTPA were detected by intention group, χ^2^ = 54.31, *p* < .001; non-intender, ambivalent nonactor, and ambivalent groups were more likely to report very light or light activity; moderately active participants were more likely to be ambivalent actors; active participants were more likely to report being successful intenders or ambivalent actors; and the very active were more likely to be disinclined actors or ambivalent nonactors. No differences existed by intention groups in key message codes mentioned, or reasons for participation (e.g., prizes). There were differences in reported impact of the Play List, χ^2^ = 26.19, *p* < .001. Non-intenders and ambivalent nonactors were less likely to report engaging in more regular PA or a new activity because of the 150 Play List, but they were more likely to report they were encouraging others to be active, to be less sedentary, or to say it had no impact.

For parsimony, only the summary results of the RM ANOVAs are reported. A significant increase occurred in overall number of 150 Play List activities tried since registration from baseline (M = 27.11 [SD = 32.30]) to follow-up (M = 31.88 [SD = 36.44]), but no differences existed by intention group. No change was reported in importance of PA or PA intentions, nor did change occur in these constructs by intention group. A change in attitudes was observed by intention group. Post-hoc tests showed ambivalent nonactors had a decline in attitudes from baseline (M = 5.47 [SD = 1.59]) to follow-up (M = 5.25 [SD = 1.58]), whereas successful intenders had an increase in attitudes from baseline (M = 5.68 [SD = 1.40]) to follow-up M = 5.87 [SD = 1.24]). Finally, a significant overall decline was observed in self-efficacy from baseline (M = 82.01 [SD = 22.03]) to follow-up (M = 80.72 [SD = 21.89] but no differences existed by intention group. The effects were very small, Cohen’s *d* range = .01–.14,

Significant differences existed in intention groups in the number of activities planned to continue, the number of new activities tried at follow-up, and the number of new activities participants plan to continue. In all cases successful intenders reported more activities than any other intention group, and there were significant differences in activities successful intenders planned to continue (M = 21.78 [SD = 28.08]), and new activities tried (M = 15.30 [SD = 18.63]), compared to non-intenders (respective M [SD] = 12.06 [18.50], 9.86 [8.34]), or ambivalent nonactors (respective M [SD] =14.28 [23.41], 9.47 [11.06]). Successful intenders also reported more new activities tried that they planned to continue (M = 8.56 [SD = 13.23]) than ambivalent nonactors (M = 4.04 [3.18]). Effect sizes were moderate, Cohen’s *d* range = .29–.47.

Because 203 participants did not report seeing any 150 Play List advertisement, the following regression analyses were conducted with the sample of 1095 who did. Table 4 shows the results of the binomial logistic regression indicating whether participants tried new activities at follow-up. Because of some very small cell sizes (i.e., *n* < 10), sex, education, and minority groups were not included in this analysis. There was a positive relationship between trying new activities and citing ‘getting active’ in responses to the key message question at baseline, higher rated experience with the 150 Play List, and higher attitudes toward 150 Play List advertisements.

**Table 4:** Tried new activities at follow-up – likelihood of saying yes compared to no

		Step 1	Step 2	Step 3
		Χ^2^ = 6.89 (*df* = 4), *p* = .14; Nagelkerke R^2^ = .009	Χ^2^ = 10.63 (*df* = 4), *p* = .005; Nagelkerke R^2^ = .023	Χ^2^ = 139.82 (*df* = 6), *p* < .001; Nagelkerke R^2^ = .195
Predictor		Exp (B)	Exp (B)	
Age		.99`	.991	.984*
LTPA	Very light or light	0.753	0.735	0.709
	Moderate	1.060	1.062	1.111
	Active	1.199	1.180	1.174
	Very active^a^	–	–	–
Mentioned 150 Play List			1.340	1.279
Mentioned ‘getting active’			1.622*	1.510
Importance				0.966
Affective attitudes				1.033
Self-efficacy				0.989
Intentions				1.113
150 Play List experience				1.643**
Ad attitudes				1.796**

^a^comparison group; *p < .01, **p < .001

Table 5 shows the results of the multinomial logistic regression predicting intention group with successful intenders compared to all other intention groups. Because there were no differences in sex, education, or minority groups by intention group, and some very small cell sizes (i.e., *n* < 10), these variables were not included in this analysis. Non-intenders and ambivalent nonactors had significantly lower baseline PA intentions than successful intenders. All intention groups, except ambivalent actors, rated their Play List experience as lower than successful intenders. Unsuccessful intenders had significantly lower attitudes toward the 150 Play List advertisements than successful intenders.

**Table 5:** Predictors of intention groups – all compared to successful intenders

		Χ^2^ = 230.82, *p* < .001; Nagelkerke R^2^ = .196					
		Non-intenders	Unsuccessful intenders	Disinclined actors	Ambivalent nonactors	Ambivalent actors	Ambivalent
Predictor		Exp (B)	Exp (B)	Exp (B)	Exp (B)	Exp (B)	Exp (B)
Age		0.965**	.967**	.990	.981**	.990	.983
LTPA	Very light or light	1.691	0.740	1.289	1.719	1.606	5.937**
	Moderate	0.408	1.027	0.512	0.531	1.010	1.258
	Active	0.489	0.888	0.537	0.507	1.051	1.047
	Very active^a^	–	–	–	–	–	–
Mentioned 150 Play List		0.594	1.015	1.876	0.884	1.210	0.652
Mentioned ‘getting active’		1.076	1.001	1.036	0.726	1.136	0.655
Importance		1.007	1.199	1.594	.973	1.005	1.058
Affective attitudes		1.060	1.034	0.808	1.149	0.983	1.064
Self-efficacy		1.018	0.991	0.995	1.007	1.003	1.012
Intentions		0.608**	0.862	1.165	0.731	0.922	0.819
150 Play List experience		0.586**	0.821	0.754*	0.677**	.903	0.645**
Ad attitudes		0.778	0.479*	0.617	0.660	.628*	0.724

^a^comparison group; * *p* < .01, **p < .001

Table 6 shows the results of the follow-up linear regressions. Similar to the baseline findings, very little variance was accounted for in any of the models. Higher rated 150 Play List experiences were significantly related to all variables. Higher attitudes toward the advertisements were significantly related to number of overall and new activities planned to continue.

**Table 6:** Descriptive statistics, Step 3 *F* -tests, full model R^2^ and coefficients for the follow-up linear regression models for number of activities from 150 Play List tried and plan to try, and number of NEW activities tried because of the Play List and new activities planned to continue

	Number of activities tried since registration	Number planning to continue^b^	Number of NEW activities tried^c^	Number of NEW activities plan to continue^c^
Descriptives ^a^	*N* = 1095, M = 32.65 (SD = 36.50)	*N* = 1007, M = 17.59 (SD = 25.32)	*N* = 807, M = 12.82 (SD = 19.14)	*N* = 714, M = 6.47 (SD = 13.04)
Predictor	F (6, 1081) = 21.39, *p* < .001; R^2^ = .13	F (6, 1081) = 15.42, *p* < .001; R^2^ = .09	F (6, 793) = 8.22, *p* < .001; R^2^ = .07	F (6, 700) = 8.76, *p* < .001; R^2^ = .09
Age	−.068	−.081	−.065	−.012
Sex	.071	.059	.032	.049
Education	.022	−.029	.017	−.030
Minority group	.015	−.008	.005	−.054
LTPA	.027	.009	.080	.060
Mentioned 150 Play List	.040	.028	.065	.065
Mentioned ‘getting active’	.030	−.003	−.013	.094*
Importance	.035	.019	.038	.101
Affective attitudes	.022	−.029	−.183**	−.043
Self-efficacy	−.004	.053	.030	−.020
Intentions	.041	.040	.067	−.046
150 Play List experience	.307**	.212**	.228**	.198**
Ad attitudes	.006	.088*	−.069	.090

* *p* < .01, **p < .001

^a^Only participants who had advertisement attitudes data were included

^b^Missing data in this variable were mean replaced; the results of an analysis with or without mean replacement did not differ

^c^because of the large amount of missing data and the nature of the question (i.e., participants may not have answered if they didn’t try new activities) the analyses were conducted without replacing the missing data

## Discussion

Media and educational campaigns have been identified as one population approach to increase PA, yet a 2012 scientific statement from the American Heart Association concluded that there is not enough established evidence regarding their efficacy and utility in health behavior change [15]. To help address this deficiency this research longitudinally examined the short-term post-campaign effects of the 150 Play List on people who registered with the campaign website. The results inform whether a year-long campaign like the 150 Play List can be effective in influencing the behavior of those registered with the campaign.

At baseline, about 54% of the sample recalled some form of advertisement that prompted participation in PA or sport; 14.5% of all participants who registered with the 150 Play List recalled the campaign and 23.6% mentioned getting active. Those who named something related to either of these reported more activities tried and more activities planned to try than those who did not name them. They were also more likely to say they had tried a new activity and planned ongoing participation. This indicates that, similar to others [5], ‘top of mind’ awareness (i.e., the first thing that comes to mind when asked about a particular topic) is likely an important factor to measure, even among people already engaged with a mass reach PA campaign. However, at follow-up, results from the current study indicate that these markers of awareness were not related to ongoing participation in terms of numbers of new activities tried or planned. Only mentioning recall of a ‘getting active’ message was positively related to the number of new activities participants planned to continue. This indicates that awareness is likely important at the beginning of campaign initiation, but other factors are necessary for ongoing participation.

Other correlates of participation at baseline were age, with younger adults more likely to plan to try more activities; but older adults (albeit with extremely small odds) were more likely to report trying new activities or planning ongoing participation. Participants with a disability were more likely to have tried a new activity compared to those not in a minority group. This may indicate that the adaptations made for each of the 150 activities on the Play List inspired some to try something new or that the 150 Play List was inclusive, irrespective of adaptations. Similarly, participants who were new Canadians were also more likely to try a new activity. Although Indigenous activities were profiled on the 150 Play List, the proportion of participants in the current research who identified as Indigenous (i.e., 2.2%, with only 1.5% at follow-up) was about half the proportion of Indigenous persons in the Canadian population (i.e., 4.3% [16]). Further efforts are needed to attract minority groups to the website portions of large scale PA campaigns. An additional finding is that participants reporting moderate LTPA or being active compared to the very active at baseline were more likely to plan ongoing participation. Thus, the 150 Play List may have been effective in increasing PA in those who do a little activity but might not meet Canada’s PA guidelines.

In terms of possible outcomes proximal to awareness, perceptions of the importance of PA or attitudes regarding PA did not change and there were small decreases in self-efficacy and intentions, but with very small effect sizes. Other researchers have reported that although self-efficacy may increase at the start of an exercise campaign, it tends to start to decrease after a few months, a finding the authors attribute to the possibility that self-efficacy declines as participants realize that long-term adherence is challenging [17, 18]. This may also be demonstrated in the current study in the optimism demonstrated at baseline in which participants planned to try an average of forty-eight 150 Play List activities, but at follow-up an average of about thirty-two activities had been tried. Though still a substantial number of activities, the level of participation (e.g., how long, at what intensity, how many times) is unknown, and participants may have found it difficult to accurately assess self-efficacy at baseline.

Further, self-efficacy was not related to participation outcomes at follow-up. However, participants who reported PA as less pleasant reported trying fewer new activities. Other significant correlates of trying new activities at follow-up were younger age, more positive reported experience with the 150 Play List, and stronger positive attitudes towards the advertisements. More positive 150 Play List experience was also related to the number of activities tried since registration, the number of new activities tried, and the number of new activities planned to continue. Thus, and not too surprisingly, ensuring a positive experience with a campaign is essential. Taken together, these results highlight the importance of a positive affective response to a PA campaign. There is strong evidence that a positive affective response while engaging in moderate PA is related to future PA [19].

The intention groups were also informative regarding campaign impacts and continuation intentions. Participants who indicated that trying a 150 Play List activity resulted in ongoing participation at baseline, and maintained this at follow-up, were older and reported a more positive 150 Play List experience. They also had higher PA intentions than non-intenders. The very active were more likely to be disinclined actors or ambivalent nonactors. Though counter to prior research [10], this finding may be due to the 150 Play List specificity in our measurement of intentions. Specifically, the very active are likely already involved in a lot of activities and, although initially attracted to the campaign, they may not have continued because they were already engaged in other activities. Non-intender, ambivalent nonactor, and ambivalent groups were more likely to report very light or light activity and moderately active participants were more likely to be ambivalent actors. Non-intender, ambivalent nonactor, and ambivalent groups were more likely to report very light or light activity and moderately active participants were more likely to be ambivalent actors. This may indicate that motivation among the ambivalent and ambivalent nonactor groups was lacking because they were not sure if they would continue to participate in 150 Play List activities. As argued by Rhodes and Rebar [20], intention can be considered as the magnitude of strength of conviction to achieve a goal. However, it is encouraging that moderately active participants were more likely to change from being not sure about ongoing participation to indicating that the activities they tried did result in ongoing participation. Thus, as already noted, the 150 Play List may have been effective in motivating somewhat active people to be more active. However, those who did not intend further participation, or remained ambivalent, were not very active commensurate with intention-based theories [21]. Non-intenders and ambivalent nonactors were more likely to report they were encouraging others to be active or taking steps to being less sedentary. Thus, even though the campaign may not have affected their PA, it may have affected their intentions to help others be active, or become motivated to reduce sedentary activity. These are important actions, independent of PA behavior.

The strengths of this research include the longitudinal follow-up (with repeated measures) of a relatively large sample size and the application of theoretical frameworks in guiding analysis and interpretation. However, several limitations should be mentioned. First, the majority of the sample were women. These results are similar to participants from the web-based “Canada on the Move” campaign that used cereal boxes to distribute ‘free’ pedometers and asked people to visit a website to ‘donate their steps to science’ [22]. Why women are more attracted to registering for web-based campaigns warrants further consideration. A second limitation is that the sample that provided survey responses are likely biased toward the 150 Play List or to PA in general. However, even among this sample, important correlates and patterns of behavior were identified. Further, although previous research has supported the use of single item measures [13] using single items could reduce reliability. The questions regarding ongoing participation were used to create the intention groups. It is possible they measure behavioral stability rather than intention. However, the response option ‘not sure’, which about 43% of participants chose at one or both time points, likely indicates participants’ questioning whether they will continue the behavior, and thus may be related to intentions*.* A final limitation is that some participants completed the survey after they had been in the campaign for several months.

## Conclusion

In conclusion, this research helps address the lack of evidence regarding the efficacy of mass reach PA campaigns. Specifically, this research reinforces the idea that ‘top of mind’ awareness should be measured. Positively, results showed that even those who report very light activity may still engage in such a campaign to help others or to reduce sedentary behaviors. The adaptations of each activity described on the website may have encouraged people with disabilities to try new activities, but it is still not clear how to attract minority groups to engage with the website portion of such campaigns. This research identifies important characteristics of who may be attracted to such a campaign and who may wish to stay engaged.

## Additional file

Additional file 1: Survey questions. A table containing all items from the baseline and follow-up surveys by question category: demographics, awareness, 150 Play List participation, Proximal HOEM constructs, Leisure-time PA, Attitudes toward advertisements. (DOCX 19 kb)

## Abbreviations

HOEM: Hierarchy of Effects Model
PA: physical activity
