# Supplementary material for: The short-term effects of a mass reach physical activity campaign: an evaluation using hierarchy of effects model and intention profiles
Source: BMC Public Health. 2018 Nov 27;18:1300. doi: 10.1186/s12889-018-6218-7 (PMC6258458; doi:10.1186/s12889-018-6218-7)
Supplement: Supplementary file 1 — Survey questions. A table containing all items from the baseline and follow-up surveys by question category: demographics, awareness, 150 Play List participation, Proximal HOEM constructs, Leisure-time PA, Attitudes toward advertisements. (DOCX 19 kb) [file 12889_2018_6218_MOESM1_ESM.docx]

Supplemental file – survey questions

| Question category | Baseline | Follow-up |
| --- | --- | --- |
| Demographics | - year born (open-ended) - sex (female, male, prefer not to answer) - education (less than high school, high school, some college, college or university degree, post degree, prefer not to answer) - self-identification with any of the following: new Canadian, visible minority, Indigenous, disabled, none of the above (check all that apply). | None |
| Awareness | - do you remember seeing any advertisements (on TV, social media, internet, and/ or newspaper) that prompted participation in any specific PA or sport program (yes/no) - If yes, “Can you describe any of the key messages?” (open-ended) | None |
| 150 Play List participation | - date registered on the website (month/day/year) - how many activities from the 150 Play List “have you tried since registration” (open-ended number) and “do you plan to try” (open-ended number). - “Did you try any NEW activities because of the 150 Play List” (yes, no), - If yes “Did any of the activities you tried on the 150 Play List result in on-going participation?” (yes, no, not sure). | - how many activities from the 150 Play List “have you tried since registration” (open-ended number) and “do you plan to continue” (open-ended number). - “did you try any NEW activities on the 150 Play List” (yes/no) - if yes “how many NEW activities did you try” (open-ended number), “how many do you plan to continue to participate in” (open-ended number), and “did any of the activities you tried on the 150 Play List result in on-going participation” (yes, no, not sure) - As a result of the ParticipACTION 150 Play List are you doing any of the following (check all that apply): Signed up to participate in a sport(s) and/or physical activity, participating in sports and/or physical activity more regularly, participating in a NEW physical activity and/or sport, encourage others to participate in physical activity and/or sport, taking steps to be less sedentary, facilitating participation of others (family, friends) in sport and/or physical activity, other, had no impact |
| Proximal HOEM constructs | - Importance “participating in PA is important to me” from 1 (not at all true) to 7 (very true) - Attitudes: “for me to achieve the recommended amount of PA is” 1 (unpleasant) to 7 (pleasant) - Self-efficacy “rate your degree of confidence in achieving the recommended amount of PA if you really wanted to” from 0 (cannot do at all) to 100 (highly certain I can do) scale - Intentions “I intend to achieve the recommended amount of PA over the next month” from 1 (not at all true) to 7 (very true) | - Importance “participating in PA is important to me” from 1 (not at all true) to 7 (very true) - Attitudes: “for me to achieve the recommended amount of PA is” 1 (unpleasant) to 7 (pleasant) - Self-efficacy “rate your degree of confidence in achieving the recommended amount of PA if you really wanted to” from 0 (cannot do at all) to 100 (highly certain I can do) scale - Intentions “I intend to achieve the recommended amount of PA over the next month” from 1 (not at all true) to 7 (very true) - “overall, how would you rate your experience with the 150 Play List?” from 1 (not very good) to 7 (very good). |
| Leisure-time PA | - Select one: very light (almost none), light (walking or nonstrenuous cycling or gardening once a week), moderate (regular activity at least once a week), active (regular activities such as intense walking more than once a week), or very active (strenuous activities several times a week). | - Select one: very light (almost none), light (walking or nonstrenuous cycling or gardening once a week), moderate (regular activity at least once a week), active (regular activities such as intense walking more than once a week), or very active (strenuous activities several times a week). |
| Attitudes toward advertisements | None | - “where did you see a 150 Play List advertisements” (check all that apply): television commercial, television vignette, online, radio, newspaper, or do not recall. - among those who recalled seeing a 150 Play List advertisement: “Thinking of the 150 Play List television and online promotions, how much do you agree or disagree with the following”: “unique and different”, “fun and cheeky”, “enjoyable to watch”, “relevant to you”, “boring”, “not realistic”, and “encouraged me to participate in the program.” from 1 (strongly disagree) to 5 (strongly agree) |

Note: HOEM = hierarchy of effects model; PA = physical activity
